# Rapid dissemination of *Francisella tularensis *and the effect of route of infection

**DOI:** 10.1186/1471-2180-8-215

**Published:** 2008-12-09

**Authors:** Sandra S Ojeda, Zheng J Wang, Chris A Mares, Tingtung A Chang, Qun Li, Elizabeth G Morris, Paul A Jerabek, Judy M Teale

**Affiliations:** 1 Department of Microbiology and Immunology, University of Texas Health Science Center at San Antonio, 7703 Floyd Curl Dr., San Antonio, TX, 78229, USA; 2 MPI Research, 54943 North Main Street, Mattawan, MI, 49071, USA; 3 Department of Radiology, University of Texas Health Science Center at San Antonio, 7703 Floyd Curl Dr., San Antonio, TX, 78229, USA; 4 Research Imaging Center, University of Texas Health Science Center at San Antonio, 7703 Floyd Curl Dr., San Antonio, TX, 78229, USA; 5 Department of Biology, University of Texas at San Antonio, One UTSA Circle San Antonio, TX, 78249, USA

## Abstract

**Background:**

*Francisella tularensis *subsp. *tularensis *is classified as a Category A bioweapon that is capable of establishing a lethal infection in humans upon inhalation of very few organisms. However, the virulence mechanisms of this organism are not well characterized. *Francisella tularensis *subsp. *novicida*, which is an equally virulent subspecies in mice, was used in concert with a microPET scanner to better understand its temporal dissemination in vivo upon intranasal infection and how such dissemination compares with other routes of infection. Adult mice were inoculated intranasally with *F. tularensis *subsp. *novicida *radiolabeled with ^64^Cu and imaged by microPET at 0.25, 2 and 20 hours post-infection.

**Results:**

^64^Cu labeled *F. tularensis *subsp. *novicida *administered intranasally or intratracheally were visualized in the respiratory tract and stomach at 0.25 hours post infection. By 20 hours, there was significant tropism to the lung compared with other tissues. In contrast, the images of radiolabeled *F. tularensis *subsp. *novicida *when administered intragastrically, intradermally, intraperitoneally and intravenouslly were more generally limited to the gastrointestinal system, site of inoculation, liver and spleen respectively. MicroPET images correlated with the biodistribution of isotope and bacterial burdens in analyzed tissues.

**Conclusion:**

Our findings suggest that Francisella has a differential tissue tropism depending on the route of entry and that the virulence of Francisella by the pulmonary route is associated with a rapid bacteremia and an early preferential tropism to the lung. In addition, the use of the microPET device allowed us to identify the cecum as a novel site of colonization of *Francisella tularensis *subsp. *novicida *in mice.

## Background

*Francisella tularensis *is a facultative intracellular pathogen that is the causative agent of tularemia. Francisella has a broad host range as it is able to infect amoeba, arthropods, rodents and higher mammalian species [1-4]. The primary replication site in humans appears to be the macrophage although other cell types have been implicated [5-7]. Infection is established through contact with infected tissues, arthropod bites, inhalation or ingestion which can lead to various clinical manifestations [3,8]. The route of infection is a key determining factor in the pathogenesis of this organism, and inhalation is the most dangerous [3,9,10]. There are two subspecies of *F. tularensis*, that are capable of causing disease in humans [9,10]. *F. tularensis *(type A) is primarily found in North America. It is the most virulent strain and is capable of causing disease with as few as 10 organisms. The highly virulent nature of this microorganism combined with its ability to be aerosolized has led to its designation as a Category A biological warfare agent by the CDC [3,11]. *F. tularensis *subsp. *holarctica *(type B) is less virulent than type A, but it is found more readily across the northern hemisphere. Type B is also the parent strain from which the important *F. tularensis *LVS was derived [12]. *F. tularensis *subsp. *novicida *is another important laboratory strain as it remains highly virulent in mice but can be studied in a BSL-2 facility allowing access to new technology such as positron emission tomography (PET).

PET has been utilized to detect cells, proteins and gene expression in humans *in vivo *[13-15]. In humans, PET has been used as a research tool for whole body imaging and is now transitioning into diagnostic tools in oncology and in the detection of Alzheimer's disease [16]. Recent advances in microPET design have led to the production of scanners that are capable of imaging rodents with high resolution [13,17,18]. Advancements in labeling techniques as well as different substrates [19,20] have allowed for cell trafficking and distribution studies [15,21,22] as well as for tracing bacteria in infections [23,24] and for tumor detection [25]. The major advantage to utilizing microPET imaging is that it is a non-invasive process with high sensitivity that allows the investigator to perform longitudinal studies on one animal rather than sacrificing several animals at various time points [16].

We have exploited the capability of small animal microPET coupled with radionuclide labeling to help us investigate the spatial and temporal biodistribution of *F. tularensis *subsp. *novicida *in vivo. Mice were infected with ^64^Cu-pyruvaldehyde-bis(N^4^-methylthiosemicarbazone) ([^64^Cu] PTSM) labeled *F. tularensis *subsp. *novicida *by different inoculation routes including intranasal, intratracheal, intragastric, intraperitoneal, intravenous and intradermal. The trafficking of labeled *F. tularensis *subsp. *novicida *was compared with the control organisms *E. coli *and *K. pneumoniae *using both microPET analysis and radionuclide biodistribution analyses. Our results indicate that *F. tularensis *subsp. *novicida *has a differential tissue tropism depending on the route of entry and that when given by the intranasal or intratracheal route has a propensity to colonize the respiratory tract rapidly after infection tending to disseminate to other target organs within a very short period of time.

## Results

### In vivo trafficking of bacteria in real time

**Figure 1:**
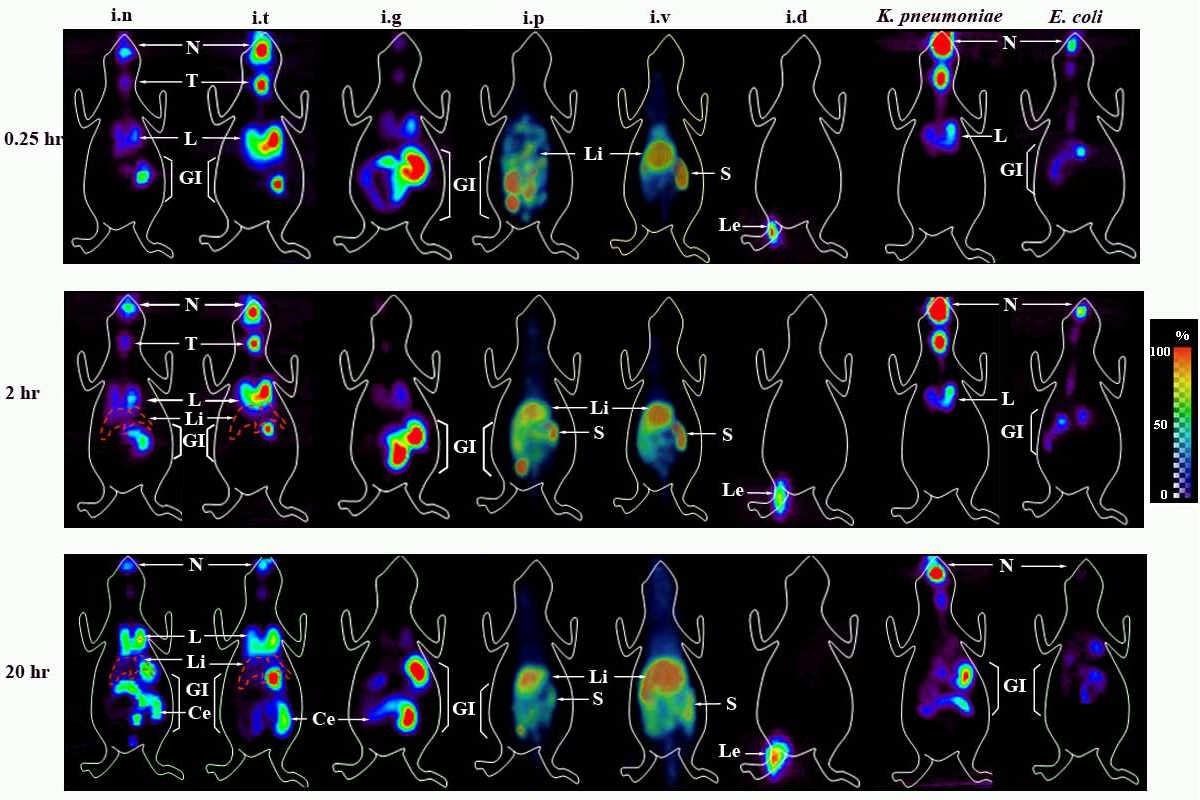
**MicroPET imaging of bacterial infection of mice**. Two-dimensional images of representative mice (ventral view) are depicted in the figure. MicroPET images were recorded over time from mice infected with either ^64^Cu labeled *F. tularensis *subsp. *novicida*, ^64^Cu labeled *K. pneumoniae *or ^64^Cu labeled *E. coli*. Mice were infected i.n, i.t, i.g, i.p, i.v and i.d with *F. tularensis *subsp. *novicida *(2 × 10^9 ^CFU/20 μl) and i.n with *K. pneumoniae *and *E. coli *using the same infection dose. Images were obtained at 0.25 hrs, 2 hrs and 20 hrs p.i. The red punctuated line represents the liver. The color scale on the right hand side of the figure is a linear scale that indicates percentage with red indicating 100% of signal and purple 0–10% or the lowest amount detected by imaging. Abbreviations: N: Nasal cavity, T: Trachea, L: Lung, GI: Gastrointestinal Tract, Li: Liver, S: Spleen, Le: Leg, Ce: Cecum.

**Figure 2:**
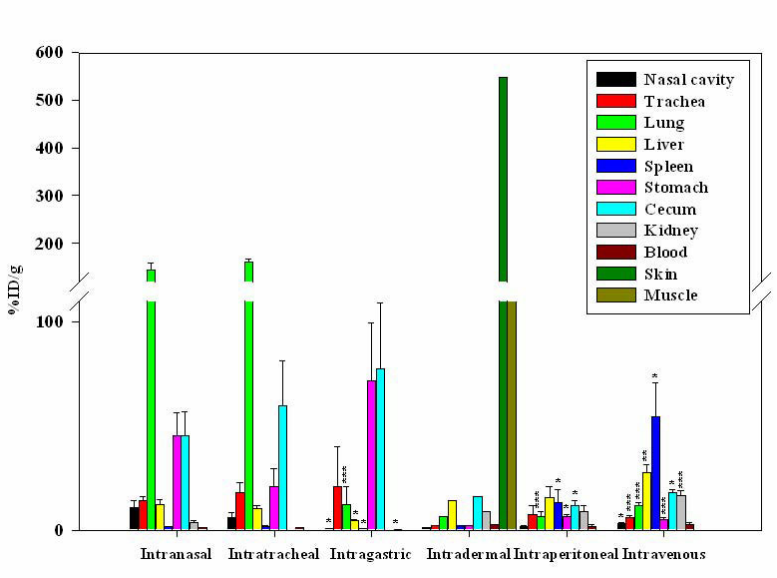
**Biodistribution of ^64^Cu labeled *F. tularensis *subsp. *novicida***. In order to confirm the observations from the microPET images the distribution of labeled bacteria was further analyzed by measuring the amount of radioactivity present in individual tissues at 20 hrs p.i. The data represent an average of 3–9 mice per route of infection and are expressed as % ID/g of tissue. The data obtained for each of the tissues sampled following i.n infection was compared statistically to the same tissue when inoculated by the i.g, i.p and i.v routes by using a two tail Student's t-test [e.g. lung (i.n) to lung (i.g)]. * = p < 0.05, ** = p < 0.01 and *** = p < 0.005. Data obtained following the i.d route was not included for the statistical analyses because it corresponded to just two biological replicates. The break in the scale was done between 110 and 120.

*F. tularensis *subsp. *novicida*, a highly virulent strain in mice, was directly labeled with ^64^Cu-PTSM so that its route of dissemination and anatomical distribution after inoculation could be traced by repetitive microPET imaging of live animals. C57BL/6 mice were infected i.n with 2 × 10^9 ^CFU/20–25 μl of bacteria and accumulated positron emission measured for a 15 min period immediately after inoculation (labeled 0.25 hr), 2 hrs and 20 hrs post-infection (p.i) by using a microPET scanner. Still images are shown in Fig. 1, and images that can be rotated 360° can be found in Additional File 1. Surprisingly, by 0.25 hr (15 min) p.i we observed radiolabel not only in the nasal cavity at the site of inoculation but also in the lung and even in the digestive tract (Fig. 1). The inoculum volume of 20–25 μl has been shown to avoid introduction into the lung [26,27], but the rapid spread was found repeatedly in multiple animals. At 2 hrs p.i labeled organisms tracked to the same organs but the amount of ^64^Cu detected by the microPET increased in the lungs as well as the GI tract suggesting an increase in the number of bacteria present in these organs. Interestingly, the cecum was usually a hot spot for labeled organisms. The images obtained 20 hrs post-infection indicated a decrease in label intensity present in the nasal cavity, an additional increase in the lung and further spread through the GI tract (Fig. 1). The recent acquisition of a FLEX Pre-Clinical Platform allowed us to obtain an X-ray computed tomography (CT) image of the skeletal structure of mice that had been previously infected by the i.p and i.v routes prior to acquire the microPET images (Additional Files 2 and 3). The advantage is that a fused image of the skeletal structure with the microPET image is obtained and in this way a better indication of the localization of the labeled organisms in vivo is possible. Additional Files 1, 2 and 3 show fused images obtained at 0.25 hrs for i.n. i.p and i.v inoculations. To further analyze isotope distribution, inoculated animals were sacrificed, and several organs harvested to measure the amount of radioisotope using an automatic well-type γ counter. The combined ex-vivo data obtained from several experiments are expressed as % input dose/g of tissue (%ID/g) (Fig. 2). The results obtained with *F. tularensis *subsp. *novicida *i.n infected animals correlate with the microPET analyses indicating the highest concentration of ^64^Cu in the lung, stomach and GI tract. Importantly, essentially all organs and tissues tested contained radioactivity suggesting widespread dissemination of Francisella by 20 hrs p.i.

To ensure that the radioactivity was indeed a measure of viable labeled bacteria, four tissues were analyzed for CFU/g of tissue at 20 hrs p.i (Additional File 6). Due to normal flora, intestinal tissues were not analyzed but Francisella is also known to colonize the liver. Consistent with radioisotope measurements in vivo and ex vivo, the lungs contained the highest bacterial burden with greater than 4–5 logs of bacteria/g of tissue compared with the bladder. The liver was also several logs lower reflective of a %ID/g that was visualized by microPET at 20 hrs with a blue/purple color indicative of lower levels of radioisotope/g. Taken together; these results indicate that levels of radioisotope reflect numbers of viable organisms. Because of the rapidity of dissemination, the presence of viable organisms in the blood was also tested and found to be present (Additional File 6).

### Viable vs. non-viable bacteria

**Figure 3:**
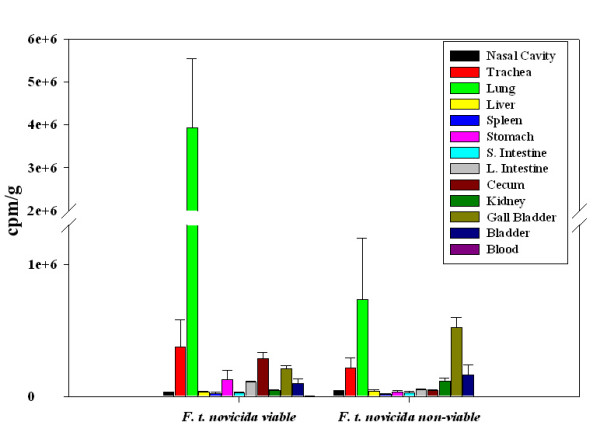
**Biodistribution of viable vs. non viable ^35^S labeled bacteria**. As a mode to compare the localization of labeled bacteria, metabolic labeling was performed for *F. tularensis *subsp. *novicida*. The data represent an average of 3 mice per treatment and are expressed as cpm/g. Viable (unfixed) labeled *F. tularensis *subsp. *novicida *is found mainly in the lungs while there was an increase in non-viable (fixed) organisms localizing primarily to the gall bladder.

To determine the effect of viability on bacterial trafficking, *F. tularensis *subsp. *novicida *metabolically labeled with ^35^S-L-methionine and ^35^S-L-cysteine was taken to a titer of 2 × 10^9 ^and then fixed in 4% paraformaldehyde. Subsequently labeled bacteria were administered to mice i.n, and the accumulation of label in various tissues was compared with that of non-fixed, viable organisms. The data are expressed as counts per minute/g (cpm/g) (Fig. 3) and indicate that the bulk of the label was present in the lungs at 20 hrs p.i when viable (unfixed) organisms were used but more evenly distributed among the tissues analyzed when non-viable (fixed) organisms were used. In addition, use of non-viable organisms resulted in a significant increase in label in the gall bladder compared with viable organisms.

Several of the tissues from mice receiving viable organisms were also tested for CFU/g of tissue (data not shown). Similar to the results obtained with ^64^Cu labeling, the lung exhibited the highest bacterial burden from all the organs tested. Viable bacteria were also detected in the liver, spleen, superficial lymph nodes, Peyer's patches, and blood correlating with the presence of isotope but at lower levels in all of these tissues.

### Effect of bacterial route of entry

**Figure 4:**
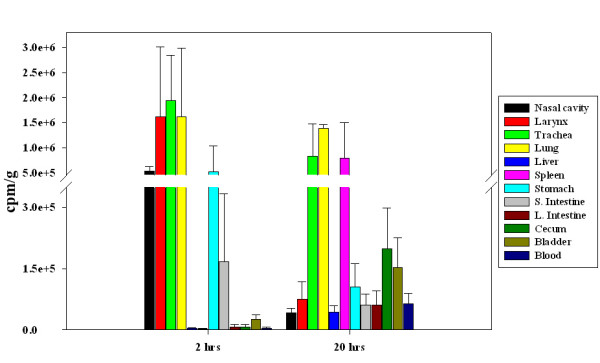
**Presence of *F. tularensis *subsp. *novicida *in the GI tract at early time points**. As a way to discard the possibility of swallowing of the inoculum during i.n and i.t infections we tested an endotracheal entubation of mice to directly deliver ^35^S labeled *F. tularensis *subsp. *novicida *into the trachea. Data shown represent an average of 3–4 mice and is given as cpm/g. Our biodistribution results showed that after 2 hrs p.i the majority of the labeled organisms were present in the lung and trachea; furthermore, a high proportion of labeled organisms were present in the stomach. In addition, after 20 hrs p.i the highest amount of labeled bacteria was present in the lung and further disseminated to other tissues including the cecum.

*Francisella tularensis *can establish infection in the host when entering through different routes. The severity of the disease differs according to the route of infection and the inhalation route is known to be the most dangerous form. Due to these facts and to the results obtained in our microPET studies following i.n infection, our laboratory decided to study *F. tularensis *subsp. *novicida *in vivo distribution following other routes of infection by which Francisella is also known to enter the host and cause disease. First, we included an important control for the i.n infection. When bacteria were administered i.n in just 20–25 μl, labeled organisms appeared in the GI tract by 0.25 hr (15 min) including the stomach, therefore it was important to control for swallowing. So in a set of experiments, mice were inoculated with the same dose of organisms (2 × 10^9 ^CFU) but by the i.t route (Fig. 1 and Additional File 4 left). The i.t inoculation was done by administering the bacterial dose at the back of the throat while holding the tongue in an extended position thus preventing swallowing as described previously [28,29]. This explains label in the head area at the site of inoculation. The results obtained were similar in that labeled organisms could be observed by microPET in the gastrointestinal tract by 0.25 hr (15 min). Similar to the i.n route, by 20 hrs the majority of label appeared in the lung and GI tract including the cecum although the relative amount of label in the lung was higher, especially at 0.25 and 2 hrs p.i. The results observed by microPET were confirmed by determining the biodistribution of label ex vivo in various tissues and the data represent an average of 3 animals after imaging (Fig. 2). To further discard the possibility of swallowing of the inoculum during i.n and i.t infections we decided to test an endotreacheal intubation of the mice to directly deliver ^35^S labeled *F. tularensis *subsp. *novicida *into the trachea. Our biodistribution data shows that after 2 hrs p.i the majority of the labeled bacteria are localized in the trachea and lung; interestingly, there is also a high proportion of labeled *F. tularensis *subsp. *novicida *present in the stomach (Fig. 4). In addition, after 20 hrs p.i the biodistribution data shows that the highest concentration of labeled bacteria appeared to be present in the lung and trachea followed by the spleen; furthermore, labeled *F. tularensis *subsp. *novicida *appeared to be present in the cecum as well (Fig. 4). These results correlate with the ones obtained by microPET, early after infection *F. tularensis *subsp. *novicida *localizes in the lung and stomach, while after 20 hrs p.i the bacterium remains in the lung and further disseminates to other tissues including the cecum. Also of relevance, *F. tularensis *is known to cause illness in humans by ingestion, through cuts in the skin, as well as systemically but with usually different clinical outcomes. Therefore, mice were also inoculated with the same dose of bacteria by the i.g, i.d, i.p and i.v routes. Following i.g administration, the majority of labeled organisms appeared in the GI tract and remained so for 20 hrs p.i (Fig. 1 and Additional File 4 right). Substantial tropism to the lung was not evident by 20 hrs. Mice inoculated by the i.d route exhibited a different pattern (Fig. 1 and Additional File 5 right and left). The vast majority of the bacteria injected appeared to remain at the site of inoculation for the initial hours of infection. Consistent with this, the distribution of label in isolated tissues indicated that the vast majority of the label was localized to the skin and muscle at 20 hrs (Fig. 2). Nonetheless, the isotope distribution still suggested that there were bacteria in the blood and several other tissues by 20 hrs although there was no preferential tropism to the lung at this early time point. In the case of mice infected by the i.p route, most of the labeled bacterium 20 hrs after infection appeared to be present in the liver as seen by microPET images with red-yellow color indicating the high concentration of bacteria in this tissue (Fig. 1), followed by the spleen and GI tract. In addition, our observations indicated that there was not a considerable amount of labeled bacteria present in the lung at this early time point (Fig. 1). Biodistribution data obtained for ^64^Cu labeled *F. tularensis *subsp. *novicida *at this time point correlates with the microPET data, showing a substantial tropism for the liver followed by the spleen that had a significantly higher %ID/g, than in the case of the i.n. infection (p < 0.05) (Fig. 2). In addition, statistical analysis showed that 20 hrs p.i, the lung, stomach and cecum showed a significantly higher %ID/g following i.n infection but not i.p infection (p < 0.005, p < 0.05 and p < 0.05 respectively). When the i.v infection was performed, we observed that 20 hrs p.i the majority of the labeled organisms were present in the liver, spleen and GI tract, seen by microPET image (Fig. 1). According to the biodistribution data obtained 20 hrs p.i, the majority of labeled *F. tularensis *subsp. *novicida *was present in the spleen and liver (Fig. 2), correlating with the microPET images (Fig. 1). Furthermore, statistical analyses showed that when comparing this infection route to the i.n route, there is a significantly higher tropism of *F. tularensis *subsp. *novicida *for spleen, liver and kidney (p < 0.05, p < 0.01 and p < 0.005 respectively) while there is a significantly lower tropism for the nasal cavity, trachea, lung, stomach and cecum (p < 0.05, p < 0.005, p < 0.005, p < 0.005 and p < 0.05 respectively) (Fig. 2).

### Effect of bacterial strain

**Figure 5:**
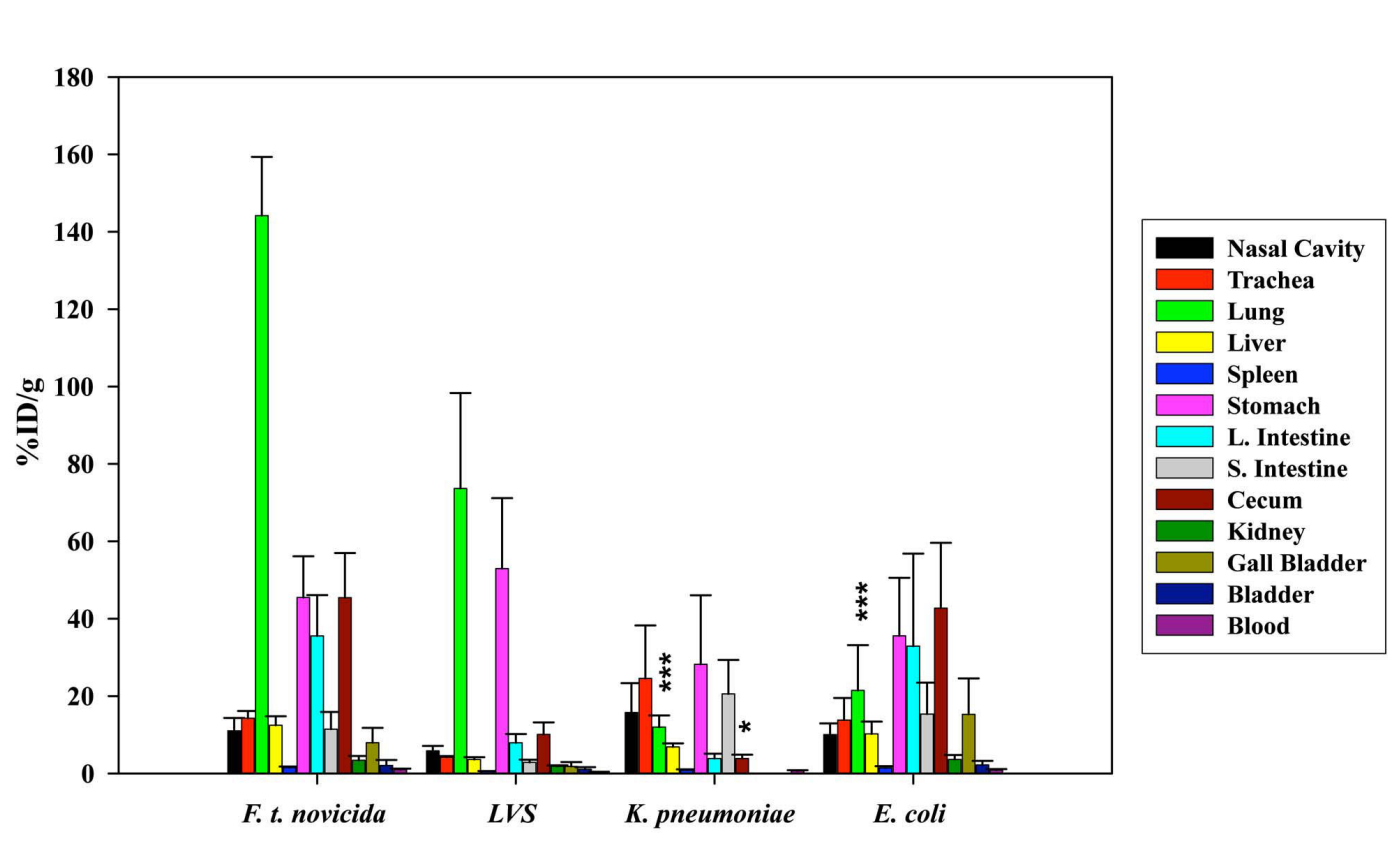
**Biodistribution of ^64^Cu labeled bacteria**. The distribution of ^64^Cu labeled *F. tularensis *subsp. *novicida *was compared to ^64^Cu labeled *K. pneumoniae*, ^64^Cu labeled *E. coli *and ^64^Cu labeled LVS in each tissue at 20 hrs pi. The data represent an average of 3 – 9 mice per infection and are given as %ID/g of tissue. The data obtained for each of the tissues sampled 20 hrs p.i from mice infected with *F. tularensis *subsp. *novicida *was compared statistically to the same tissue when inoculated with ^64^Cu labeled *K. pneumoniae *and *E. coli *at 20 hrs p.i by using a two tail Student's t-test [e.g. lung (i.n) to lung (i.g)]. * = p < 0.05, ** = p < 0.01 and *** = p < 0.005.

To test the potential effects of bacterial strains on dissemination, two different bacterial strains, *E. coli *and *K. pneumoniae*, were labeled with ^64^Cu-PTSM and administered i.n at the same dose of 2 × 10^9 ^CFU/20 μl used for *F. tularensis *subsp. *novicida*. By both microPET analysis (Fig. 1) and ex vivo isotope distribution at 20 h (Fig. 5), *E. coli *exhibited the highest percentage of radioisotope in the digestive tract and a barely detectable percentage in the lung that was significantly lower than in the case of *F. tularensis *subsp. *novicida *infected mice (p < 0.005) (Fig. 5). In the case of *K. pneumoniae *infection (Fig. 1), another pulmonary pathogen, a larger percentage of labeled bacteria remained in the nasal cavity and less in the lung compared with *F. tularensis *subsp. *novicida *at all time points tested. Furthermore, our biodistribution data showed that by 20 hrs p.i the %ID/g obtained in the lung for *K. pneumoniae *infected mice are significantly lower than for *F. tularensis *subsp. *novicida *infected mice (p < 0.005). In addition, spread of bacteria to the digestive tract appeared to be somewhat delayed compared with *F. tularensis *subsp. *novicida*. Interestingly, *F. tularensis *subsp. *novicida *shows a significantly higher tropism for cecum when compared to *K. pneumoniae *(p < 0.05) but not with *E. coli *(Fig. 5). We also labeled LVS with ^64^Cu, infected mice by the i.n route and imaged them by MicroPET (data not shown). The results were similar to those obtained with i.n inoculation of *F. tularensis *subsp. *novicida *showing higher levels in the lung and GI tract. The biodistribution data is shown in Fig. 5.

### Isolation of *Francisella tularensis *subsp. *novicida *from gastrointestinal tract

**Figure 6:**
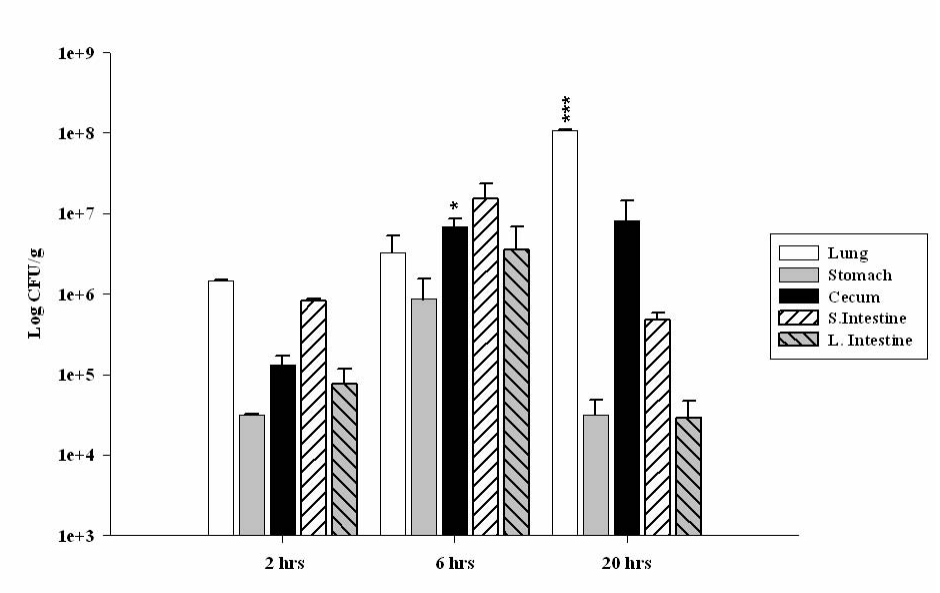
**Colonization of various tissues by *F. tularensis *subsp. *novicida***. In order to determine the presence of *F. tularensis *subsp. *novicida *in the GI tract after i.n infection a Rif^+ ^resistant strain was generated. Data represent an average of 3 mice per time point and are given as Log CFU/g of tissue. Lung, stomach, cecum, small intestine and large intestine were harvested at 2 hrs, 6 hrs and 20 hrs p.i and CFU were determined. The data obtained for each of the tissues sampled 2 hrs p.i was compared statistically by using a two tail Student's t-test to the data obtained 6 hrs and 20 hrs p.i. * = p < 0.05, ** = p < 0.01 and *** = p < 0.005.

Our microPET results show a persistent localization of *F. tularensis *subsp. *novicida *in the GI tract. To test the presence of viable organisms in these tissues, we infected mice i.n with a *F. tularensis *subsp. *novicida *Rif^+ ^strain and determined the CFU in multiple tissues at 2 hrs, 6 hrs and 20 hrs p.i. We isolated *F. tularensis *subsp. *novicida *from lung, stomach, large intestine, small intestine and cecum as early as 2 hrs p.i (Fig. 6). The results obtained showed that the number of organisms isolated from lung, stomach, large intestine, small intestine and cecum increased 6 hrs after infection although it was significant only for cecum (p < 0.05). At 20 hrs after infection, the number of CFU isolated from the stomach, large intestine and small intestine showed a decrease, which was not statistically significant. However, the number of bacteria isolated from the lung at 20 hrs was significantly increased (p < 0.005), while for the cecum the CFU remained about the same (Fig. 6).

### Presence of Francisella in blood at a lower dose

**Figure 7:**
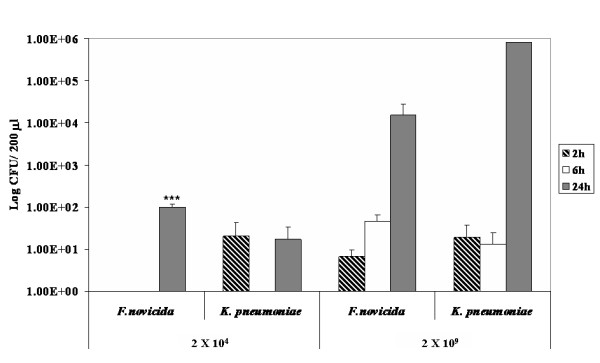
***F. tularensis *subsp. *novicida *and *K. pneumoniae *are found in blood of infected mice at early time points**. In order to determine the presence of *F. tularensis *subsp. *novicida *as well as *K. pneumoniae *in blood at early time points mice were infected with 2 × 10^4 ^CFU/20 μl and 2 × 10^9 ^CFU/20 μl. Data correspond to the average of 4 mice per infectious agent and dose and are given as Log CFU/200 μl. Mice were sacrificed 2 hrs, 6 hrs and 24 hrs pi, blood was plated and CFU were counted. The data obtained from mice infected with *F. tularensis *subsp. *novicida *at each time point and each bacterial dose was compared statistically by using a two tail Student's t-test to the data obtained from mice infected with *K. pneumoniae*. * = p < 0.05, ** = p < 0.01 and *** = p < 0.005.

As viable organisms were found in the blood by 24 hrs, it was surmised that Francisella is capable of creating an unusually rapid bacteremia. However, the microPET studies required a high dose of labeled organisms for image detection. Therefore, it was important to determine if mice receiving a lower dose would also exhibit viable organisms in the blood. For this analysis, an i.n dose of 2 × 10^4 ^CFU/20 μl was given which is comparable to the dose used in other published studies with Francisella [30]. The number of CFU/200 μl present in the blood of mice infected with 2 × 10^4 ^CFU/20 μl of *F. tularensis *subsp. *novicida *was compared with that of mice receiving the higher dose used in the imaging studies (2 × 10^9 ^CFU/20 μl). Bacteria were found in blood of mice infected with the higher bacterial dose as early as 2 hrs p.i and increased sequentially at 6 hrs and 24 hrs (Fig. 7). In mice receiving the lower dose, bacteria were detected as early as 24 hrs p.i consistent with rapid entry into the bloodstream (Fig. 7). The same experiments were performed with high and low doses of *K. pneumoniae*. The results indicated that *K. pneumoniae *is also detectable in the blood at 24 hrs p.i at significantly lower levels than *F. tularensis *subsp. *novicida *when using the low infection dose (p < 0.005). On the other hand, when comparing the presence of bacteria in blood using a higher infection dose at 24 hrs p.i, *K. pneumoniae *was present at higher levels but this difference was not statistically significant (Fig. 7).

## Discussion

Several studies have been published to examine small animal infection models using in vivo bioluminescence imaging (BLI) to track the course of infection or determine the efficacy of anti-microbial therapies [31-49]. As reviewed previously [35], there are many advantages to in vivo imaging which include the ability to perform longitudinal studies with the same animal, identifying novel sites of infection such as the cecum [48] and gall bladder [42], and increased information per animal allowing for the use of fewer animals. One of the disadvantages of BLI is the inability to accurately localize the focal point of bioluminescence within the living animal as bioluminescence produces only two dimensional data, and the spatial resolution is limited [35]. In addition, the light is significantly attenuated per cm of tissue and scattered by fur and overlying tissues further complicating the data generated [35]. The use of microPET overcomes these disadvantages providing high resolution data that are three dimensional [16]. Thus, the entire depth of the body can be measured and specific organs visualized, particularly when combined with software that enables rotation of the image, and X-ray computed tomography (CT) that reveals the skeletal structure. Unfortunately, the CT capability only became available at the very end of our experiments. In addition, the high resolution of the microPET allows for accurate determination of radioactivity within user defined areas of interest. Using these measurements, both the CFU and the % of input bacteria can be calculated without sacrificing the animal and performing labor intensive bacterial burden assays in order to track infection processes or assess therapeutic modalities. Moreover, microPET is more easily transitioned to the clinic as compared with BLI. The studies described herein used microPET technology in addition to traditional labeling techniques to assess the progressive spread of *F. tularensis *subsp. *novicida *during the initial hours of infection to explore mechanisms associated with its extreme virulence in mice.

When multiple mice were infected with radiolabeled *F. tularensis *subsp. *novicida *by inhalation followed by imaging, there was rapid widespread dissemination of organisms from the upper respiratory tract to the lung despite low inoculum volumes known to avoid direct introduction into the lung [50]. By 15 min, labeled organisms were found in the lung and stomach with further spread through the GI tract by 20 hrs. Similar results were obtained with mice infected by the i.t route using two different protocols indicating that swallowing was unlikely to be involved in the rapid dissemination to the GI tract. By 20 hrs after pulmonary infection, radioactivity substantially decreased in the upper respiratory tract. Activity in the lung was maintained and in several animals, accumulated to a greater degree suggesting an early tropism. This was confirmed by the distribution of radioisotope post necropsy where the highest levels of label were consistently found in the lung at 20 hrs. Comparable results were obtained when *F. tularensis *LVS was inoculated i.n. at the same dose suggesting similar dissemination patterns in Francisella. Similarly, bacterial burden assays have previously shown that mice intranasally infected with a low bacterial dose of Francisella show a relatively high bacterial burden in lung as early as 1 day post-infection [51,52]. Therefore, regardless of the inoculum dose, Francisella has a high tropism for the lung when infected through the aerosol route.

Bacterial burden assays correlated with the imaging and biodistribution data. A Rif^+ ^strain with equivalent virulence allowed assessment of viable bacteria in the GI tract. The results obtained show that there is a wide dissemination of bacteria as early as 2 hrs after i.n. infection, and CFU could be isolated from multiple organs including lung, stomach, small intestine, cecum and large intestine. It has been shown previously that Francisella can cause a typhoid-like disease [9] and survive the harsh environment of the GI tract in mice [53]. However, the rapidity of spread to the gut after i.n infection was unexpected. One possible explanation of the rapid dissemination to the GI tract in our model could involve a hematogenous and/or lymphatogenous way through the Nasal Associated Lymphoid Tissue (NALT). It has been previously reported [54] that the NALT is composed of mainly B cells, to a lesser extent T cells and a small proportion of macrophages (CD11b^+ ^cells), and that the percentages of these cell types observed in NALT are similar to the percentages observed in spleen [54]. Since it is known that Francisella survives and replicates inside macrophages, a possible way of dissemination from the nasal passages could be by macrophages present in the NALT acting as 'Trojan horses', assuring the protection from the immune system and facilitating a rapid dissemination to other tissues. This has been observed for *Salmonella typhimurium *by trafficking through T, B cells and macrophages [55,56].

One interesting aspect of working with microPET technology is the possibility to identify novel sites of infection. In our case, these experiments allowed us to identify the concentration of *F. tularensis *subsp. *novicida *in the cecum, which has not been described previously. Several studies using different organisms known to invade the gut including *Citrobacter rodentium*, *Helicobacter pylori *and *Burkholderia cepacia *appear to colonize the cecum [38,48,57]. In addition, it has been previously reported that intranasally delivered *S. typhimurium *in swine is present in cecum as early as 3 hrs p.i [58]. The cecum, particularly the cecal patch, contain lymphoid tissue that could play an important role in immunity and possibly further bacterial dissemination. This aspect is currently under study in our laboratory.

*K. pneumoniae*, another pulmonary pathogen, was used for comparison as was a gut associated *E. coli *clinical isolate, and both were administered i.n. Interestingly, *K. pneumoniae *in contrast to *F. tularensis *subsp. *novicida *exhibited little or no dissemination to the stomach during the first two hours and was mainly confined to the nasal cavity and trachea. Even at 20 hrs, the largest % of label was still present in the nasal cavity although trafficking to the GI tract was apparent by then. Interestingly, at these early time points, the lung was not the major focus of infection. In the case of *E. coli*, organisms at 20 hrs were mainly confined to the GI tract. The results indicate distinct distribution patterns of pathogenic bacteria during the first hours of infection when mice are given the same number of organisms and by the identical route. Moreover, the early tropism to the lung in the case of Francisella was dependent upon viable organisms as non-viable, biosynthetically ^35^S labeled bacteria failed to preferentially traffic to the lung at 20 hrs compared to the viable (unfixed) labeled bacteria. More of the non-viable organisms appeared to traffic to the gall bladder as compared to the viable labeled bacteria in this tissue at the same time point. This suggests that the viable organisms begin colonizing the lung rapidly.

Initially, we thought that the extreme rapidity of dissemination was associated with the ability of Francisella to enter the bloodstream quickly as CFU could be detected in the blood at 20 hrs p.i. However, one of the disadvantages of in vivo imaging in general is the requirement to use large doses of organisms in order to visualize the images. Therefore, we questioned if more physiological doses of 10^3 ^or 10^4 ^organisms would result in a detectable bacteremia. And in fact, even at the lower doses, clear evidence of viable bacteria were found by 20 hrs supporting this contention, and likely explaining the presence of radioactivity in essentially all tissues analyzed by both labeling techniques. However, we then tested *K. pneumoniae *for its ability to establish a bacteremia at these early time points. Significantly, *K. pneumoniae *at both high and low doses entered the blood stream, and if anything, at increased levels with detection in some mice at earlier time points. Despite this, *K. pneumoniae *infected mice did not appear overtly ill whereas *F. tularensis *subsp. *novicida *mice did as judged by pilo-erection, lethargy, and hunched posture. Interestingly, *K. pneumoniae *maintained the bulk of the inoculum in the nasal cavity in contrast to *F. tularensis *subsp. *novicida*, and it is possible that this is an important site for entrance to the blood. In fact, bacteremia has been reported in tularemia [10,59] and the presence of *F. tularensis *LVS as well as SchuS4 strains in blood early after i.d infection has been described [60]. Nonetheless, the extreme virulence of *F. tularensis *subsp. *novicida *in mice may not be attributed solely to blood vasculature ingress.

*Francisella tularensis *is a bacterium that can adapt to many different environments including soil, ground water, and growth in amoeba [11]. It also replicates intracellularly within humans and a variety of other species causing significant morbidity and mortality. It contains a number of genes that facilitate survival under a variety of conditions [61]. Correlative to this, Francisella is known to cause infection in humans by inhalation, ingestion, eye contact, insect bites, and cuts in the skin [3,9]. Interestingly, the pulmonary route causes the most severe infections with substantial mortality, although other routes of infection have been known to develop into severe infections but with less frequency [9]. Therefore, other routes of infection were tested using the same number of bacteria. With oral infection (i.g), the majority of radiolabeled Francisella trafficked and remained in the GI tract through 20 hrs p.i. After an i.d injection during this same time period, most of the organisms remained in the skin and muscle tissue. Both oral and i.d (cuts in skin) routes cause illness, but usually less severe in patients, and it is possible that this alteration in trafficking is involved in the decreased severity. Mice were also infected by the i.p and i.v routes. In both cases, the majority of the bacteria were localized to the liver and spleen and to a lesser extent to the GI tract and the lung. Comparing the biodistribution data obtained at 20 hrs p.i among all of the infection routes, the results suggest that the numbers of *F. tularensis *subsp. *novicida *bacteria present in mice after i.n and i.t infection are higher in the lung than in mice infected through any of the other infection routes tested despite the same infection doses. Several studies have used LVS to examine various routes of inoculation, mostly at later time points of infection to study colonization [53,62,63]. However, KuoLee et al. [53], reported CFU in lung and GI tract at 1 day p.i following a dose of 10^8 ^CFU of LVS, consistent with our findings. In addition, Woolard et al. [63] used an i.d dose of 10^5 ^CFU of LVS and showed barely detectable CFU in the lung and spleen at 1 day consistent with our radiodistribution data at 20 hrs p.i. We speculate that the different trafficking patterns are due, in fact by differences in the innate immune response mounted depending on the initial tissue impacted as suggested by recent studies of organ specific immunity [64-69]. We and others have found evidence of a delay in the innate immune response following i.n infection, indicated by the absence of pro-inflammatory cytokines and chemokines early after infection (6 hrs p.i – 48 hrs p.i) and beginning to increase only after 72 hrs p.i [70,71]. We propose that the acute virulence associated with inhaled Francisella is its extremely rapid colonization of the lung leading to pulmonary failure and eventual multiple organ failure before an effective immune response can be elicited. To test this, future studies will use microPET with CT capabilities together with radiolabeled antibodies to both bacteria and distinct immune cell subsets so that continued dissemination, increases in bacterial biomass, and the disease process can be evaluated at later time points.

## Conclusion

By using various labeling techniques, imaging, and bacterial counting, we conclude that Francisella rapidly disseminates within hours to multiple tissues regardless of the route of infection with the possible exception of the intradermal route. However, the route of infection alters the trafficking patterns. In the case of the pulmonary route, which is the most dangerous, the bacteria rapidly traffic to the lung and throughout the GI tract but by 20 hrs appears to preferentially colonize the lung indicating an early tropism to this organ. We speculate that the mode of transmission alters the severity of the disease because of documented differences in organ specific immunity.

## Methods

### Bacterial strains and culture media

*Francisella tularensis *subsp. *novicida *strain U112 was obtained from Dr. Bernard Arulanandam (UTSA) through Dr. Fran Nano (University of Victoria). It was grown in Tripticase Soy Agar/Broth (Becton Dickinson), supplemented with 0.1% cysteine (TSAcys). *Escherichia coli *(clinical isolate) was obtained from Dr. Stephen Mattingly (UTHSCSA) and was grown in Luria Bertani Agar (LB medium). *Klebsiella pneumoniae *(ATCC # 43816) was obtained from Dr. Peter Dube (UTHSCSA) and was grown in LB. Bacteria were resuspended in broth and then taken to a titer of 2 × 10^9 ^colony forming units (CFU)/300 μl.

### Radiolabeling of bacteria with [^64^Cu] PTSM

Resuspended bacteria were added to 400 μCi [^64^Cu] PTSM and incubated in a water bath at 42°C for 1 hr. [^64^Cu] was conjugated to PTSM as previously described [72]. Radiolabeled bacteria were centrifuged at 15000 g for 4 min. Supernatants were removed, and both pellets and supernatants were measured for radioactivity to determine radiolabeling efficiency of the bacteria. Preliminary radiolabeling studies were performed using different bacterial doses ranging from 10^3 ^to 10^9 ^CFU/300 μl. It was determined that the concentration of 10^9^/300 μl was most efficiently labeled and that doses of 10^9 ^labeled organisms were required for visualization by microPET. In addition, control experiments were performed to determine the effect of labeling on viability of the bacteria with time. It was found that there was no change in the viability of the organisms after 90 min of labeling indicating a lack of toxicity associated with the labeling method. In addition, the potential leakage of label was determined by incubating labeled and washed organisms for extended periods. After incubation, bacteria were washed again, and the amount of label in the supernatant and pellets determined at 24 hrs. 79.5 +/- 2.1% of the label remained in the pellet indicating the stability of the labeling process. In addition, bacteria viability post-labeling was checked at 90 min, 24 hrs and 48 hrs post-infection and was found to be 114.5%, 43.8% and 57.4% respectively. Labeled organisms were resuspended in sterile 1× phosphate-buffered saline (PBS) for inoculation of mice.

### *In vivo *bacterial biodistribution

Labeled bacteria pellets were resuspended in 30 μl or 50 μl of sterile 1× PBS to a final concentration of 2 × 10^9 ^CFU/20–25 μl intranasally (i.n) or 2 × 10^9 ^CFU/50 μl intradermal (i.d). C57BL/6 females 6–8 wk old were anesthetized with vaporized isofluorane for the initial experiments and later by intramuscular injection of 100 μl of ketamine-xylazine mixture containing (30 mg/mL ketamine, 4 mg/mL xylazine in 1× PBS). No apparent differences in images were observed between the two methods of anesthesia. Mice were then i.n inoculated with 10–12.5 μl of bacterial suspension in each nostril drop by drop thus allowing the mice to slowly inhale the inoculum. Intradermal inoculations were done by injecting 50 μl of 2 × 10^9 ^CFU in the right leg of mice. A total of fifteen mice were inoculated i.n, nine with *F. tularensis *subsp. *novicida*, three more with *E. coli *and three with *K. penumoniae*. Additional routes of inoculation included intratracheal (i.t), intragastric (i.g), intraperitoneal (i.p) and intravenous (i.v) inoculations of ^64^Cu labeled *F. tularensis *subsp. *novicida *using 2 × 10^9 ^CFU/50 μl for the i.t and i.v infections and 2 × 10^9 ^CFU/100 μl for the i.g and i.p infections; three mice were inoculated with *F. tularensis *subsp. *novicida *for each of the routes. The i.t infection was performed according to the procedure of Rubins et al. (40). Briefly, mice were anesthetized with vaporized isofluorane, suspended in an upright position, and the tongue was extended outwards and towards the side of the mouth with forceps. The inoculum was then delivered by pipette to the back of the throat while covering the nose. This procedure has been shown to prevent swallowing with the vast majority of the inoculum delivered to the trachea and lungs [28,29]. The i.g infection was performed by using a gavage needle to directly deliver the inoculum into the stomach, while the intraperitoneal infection was accomplished by injecting 100 μl of the inoculum with a 23-gauge needle directly in the right bottom quadrant of the peritoneum. Finally, the intravenous infection was achieved by injecting 50 μl of the inoculum in the mice tail vein with a 28-gauge needle. All animals were imaged for 15–20 min, immediately after infection (labeled 0.25 hr) and at 2 and 20 hrs by using a microPET-R4 rodent scanner (Concorde Microsystems, Knoxville, TN, USA). MicroPET provides a 10 cm by 8 cm field of view with a reconstructed resolution of 2.25 mm in the central 40 mm of the field of view. Images are reconstructed using Fourier re-binning followed by two-dimensional filtered backprojection. Towards the end of our studies, a FLEX Pre-Clinical Platform (Gamma-Medica-Ideas, Northridge, CA) was acquired; this allowed the acquisition of an X-ray computed tomography (CT) image permitting the visualization of the skeletal structure of the mice being analyzed. The FLEX pre-Clinical Platform CT device provides an 8.7 cm field of view, fly acquisition, 2 × 2 binning, and 256 projections, with a spatial resolution of ~100 μm. This procedure was performed for 1 min prior to acquire the microPET image. We made use of this device to image mice that had been previously infected with ^64^Cu labeled *F. tularensis *subsp. *novicida *by the i.n, i.p and i.v routes of infection.

Once the mice had been scanned for the last time point, they were sacrificed and various tissues (as shown in Fig 2) were harvested in order to quantify the amount of ^64^Cu still present in them by using an automatic well-type gamma counter (γ 8000 Beckman Coulter Fullerton, CA). Finally, some of the tissues harvested from *F. tularensis *subsp. *novicida *infected animals were weighed, homogenized in 1× PBS, serially diluted, and plated on TSAcys (Becton Dickinson) to determine CFU/g of tissue in the i.n infected mice. All the experimental procedures were in compliance with federal guidelines and the institutional animal care and use committee.

### Isolation of rifampicin resistant *Francisella tularensis *subsp. *novicida *from the gastrointestinal (GI) tract

In order to test for CFU in the GI tract, several rifampicin resistant *F. tularensis *subsp. *novicida *(*F. tularensis *subsp. *novicida *Rif^+^) isolates were generated and tested for virulence. *F. tularensis *subsp. *novicida *was grown in a liquid culture in tripticase soy broth (TSB) supplemented with 0.1% L-cysteine with agitation at 37°C overnight (ON). 1 mL of culture was then centrifuged at 13,000 rpm for 4 min; the cell pellet was resuspended in 100 μl of 1× sterile PBS and plated on TSAcys plates containing 200 μg/mL rifampicin. Rif^+ ^bacteria were streaked again on TSAcys plates containing 200 μg/mL rifampicin. Colonies that grew better were then streaked again on TSAcys plates containing 30 μg/mL rifampicin. A Rif^+ ^isolate that was shown to have equivalent virulence to the wt strain (LD_50 _10) was then used in subsequent studies. For analyses of the GI tract, nine C57BL/6 females 6–8 wk old were anesthetized and i.n inoculated with 2 × 10^9 ^CFU/20 μl of *F. tularensis subsp. novicida *Rif^+ ^bacterial suspension. At serial time points, mice were sacrificed after anesthesia using a mixture of ketamine-xylazine. Lung, stomach, large intestine, small intestine and cecum were collected and organs homogenized; serial dilutions were made and plated on TSAcys plates containing 30 μg/mL rifampicin.

### Metabolic labeling of bacteria with ^35^S

*F. tularensis *subsp. *novicida *cultures (50 mL) were incubated at 37°C for 2 hrs with agitation. Bacterial cultures were then centrifuged, supernatants were removed and cell pellets were washed twice with either TSB or LB liquid medium. Bacteria were re-suspended in 50 mL DMEM medium without L-glutamine, L-methionine and L-cysteine (MP Biomedicals) and incubated at 37°C for 15 min with agitation. Subsequently, 230 μCi of ^35^S were added to each bacterial culture and incubated for an additional 2 hrs. Bacterial cultures were then concentrated and the amount of ^35^S present in the cell pellets and supernatants were measured to determine efficiency of labeling. Once the bacteria were labeled, serial dilutions were prepared and plated on TSAcys to determine CFU.

### Biodistribution of labeled bacteria with ^35^S

Labeled bacterial pellets were resuspended in 1× PBS to a final concentration of 2 × 10^9 ^CFU. Mice (C57BL/6 6–8 wk old) were anesthetized and inoculated i.n with 20–25 μl of the bacterial suspension and inoculated i.t with 50 μl of inoculum. In order to infect mice by the i.t route an endotracheal intubation was performed by using a BioLite small animal intubation system (BioTex, Inc., TX). Briefly, mice were anesthetized by intramuscular injection of ketamine-xylazine as previously described. Subsequently mice were suspended from the incisor wire on the intubation stand, followed by the tracheal intubation with a flexible intravenous catheter over a fiber-optic laser attached to an illuminator that allows a better visualization of the oralpharyngeal cavity. Once inside the trachea, the fiberoptic was drawn out and a 1 mL syringe was plugged to the catheter, and the inculum was delivered to the mouse. This method of inoculation allowed us to avoid any accidental swallowing of the inoculum.

After infection, mice were sacrificed at 20 hrs p.i in the case of i.n infected mice and at 1–2 hrs and 20 hrs post-inoculation in the case of i.t infected mice, various tissues were removed, and the amount of ^35^S in each tissue was determined by using a multi-purpose scintillation counter (LS6500 Beckman Coulter Fullerton, CA). In addition, several tissues were weighed, homogenized in 1× PBS, serially diluted, and plated to determine CFU/g of tissue in the case of the i.n infected mice.

## Authors' contributions

SSO carried out the imaging studies, including biodistribution, metabolic labeling of the bacterium, bacterial burden assays, data analysis and writing of the manuscript. ZJW carried out the imaging studies including bacterial radiolabeling and data analysis. CAM carried out the imaging studies, including biodistribution, metabolic labeling of the bacterium and bacterial burden assays. TC participated in the imaging data analysis. LQ participated in the imaging studies, including biodistribution, metabolic labeling of the bacterium and bacterial burden assays. EGM participated in the imaging studies, including biodistribution, metabolic labeling of the bacterium and bacterial burden assays. PAJ helped supervise the use of the MicroPET. JMT conceived the study, participated in its design and coordination, and has been involved in writing and critically reviewing the manuscript.

## Supplementary Material

Additional file 1 **Movie representing in vivo localization of**^**64**^**Cu labeled*****F. tularensis *****subsp. *novicida *following i.n infection**. MicroPET and CT video of a mouse infected i.n with ^64^Cu labeled *F. tularensis *subsp. *novicida *0.25 hrs p.i. The mouse shown in this movie corresponds to one different than the one depicted in Figure 1. Click here for file

Additional file 2 **Movie representing in vivo localization of**^64^**Cu labeled *F. tularensis *subsp. *novicida *following i.p infection**. MicroPET and CT video of a mouse infected i.p with ^64^Cu labeled *F. tularensis *subsp. *novicida *0.25 hrs p.i. The mouse shown in this movie corresponds to one different than the one depicted in Figure 1. Click here for file

Additional file 3 **Movie representing in vivo localization of**^**64**^**Cu labeled *F. tularensis *subsp. *novicida *following i.v infection**. MicroPET and CT video of a mouse infected i.v with ^64^Cu labeled *F. tularensis *subsp. *novicida *0.25 hrs p.i. The mouse shown in this movie corresponds to one different than the one depicted in Figure 1. Click here for file

Additional file 4 **Movie representing in vivo localization of**^**64**^**Cu labeled *F. tularensis *subsp. *novicida *following i.t and i.g infections**. MicroPET video of two mice, one infected i.t with ^64^Cu labeled *F. tularensis *subsp. *novicida *at 0.25 hrs p.i (left when the image is still) and another one infected i.g with ^64^Cu labeled *F. tularensis *subsp. *novicida *0.25 hrs p.i (right when the image is still). The mice shown in this movie correspond to mice different than the ones depicted in Figure 1. Click here for file

Additional file 5 **Movie representing in vivo localization of**^**64**^**Cu labeled *F. tularensis *subsp. *novicida *following i.d infection**. MicroPET video of two mice infected i.d with ^64^Cu labeled *F. tularensis *subsp. *novicida *0.25 hrs p.i (right and left). One of the mice shown in this movie corresponds to the one depicted in Figure 1 while the other mouse corresponds to another one imaged at the same time. Click here for file

Additional file 6 **Graph representing bacterial load following i.n infection with**^**64**^**Cu labeled *F. tularensis *subsp. *novicida***. In order to determine the presence of viable ^64^Cu labeled *F. tularensis *subsp. *novicida *in different tissues after infection, bacterial burden assays were performed. Data represent the mean with range of two mice and are given as Log CFU/g of tissue. Lung, liver, bladder and blood were harvested at 20 hrs p.i. ^64^Cu labeled *F. tularensis *subsp. *novicida *was isolated from all the tissues analyzed at this time point. Click here for file
