# Supplementary figures and images for: Rapid dissemination of Francisella tularensis and the effect of route of infection
Source: BMC Microbiol. 2008 Dec 9;8:215. doi: 10.1186/1471-2180-8-215 (PMC2651876; doi:10.1186/1471-2180-8-215)

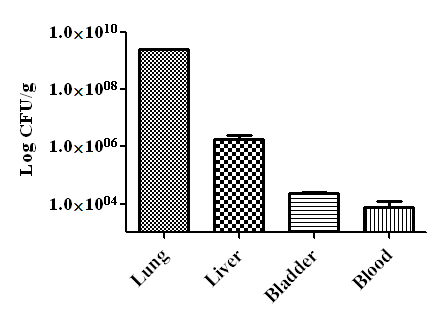

Supplement: Additional file 6 — Graph representing bacterial load following i.n infection with64Cu labeled F. tularensis subsp. novicida. In order to determine the presence of viable 64Cu labeled F. tularensis subsp. novicida in different tissues after infection, bacterial burden assays were performed. Data represent the mean with range of two mice and are given as Log CFU/g of tissue. Lung, liver, bladder and blood were harvested at 20 hrs p.i. 64Cu labeled F. tularensis subsp. novicida was isolated from all the tissues analyzed at this time point. [file 1471-2180-8-215-S6.tiff]
